# RIGulation of STING expression: at the crossroads of viral RNA and DNA sensing pathways

**DOI:** 10.14800/ics.1491

**Published:** 2017-01-01

**Authors:** Yiliu Liu, Rongtuan Lin, David Olagnier

**Affiliations:** 1 Lady Davis Institute-Jewish General Hospital, McGill University, Montreal, H3T 1E2 CANADA; 2 Department of Medicine, McGill University, Montreal, H3T 1E2 CANADA; 3 Department of Microbiology & Immunology, McGill University, Montreal, H3T 1E2 CANADA; 4 Department of Biomedicine, Aarhus Research Center for Innate Immunology, Aarhus University, Aarhus, 8000 Aarhus C DENMARK

**Keywords:** RIG-I, MAVS, cGAS, STING, antiviral response, innate immunity, type I IFN, HSV, 5′pppRNA, RIG-I agonist

## Abstract

The innate immune sensing of pathogens is important for host to mount defensive responses. STING has emerged in recent years as a critical signaling adaptor in the immune response to cytosolic DNA and RNA derived from pathogens. Liu *et al.* (2016) demonstrate that the RIG-I-dependent RNA sensing signaling induces STING expression via a TNF-α and IFN-α synergy. The up-regulation of STING is vital for 5′pppRNA restriction of HSV, a DNA virus that infects humans and causes herpes, *in vitro* and *in vivo*. This study provides new insights into the cross talk between DNA and RNA pathogen-sensing systems via the control of STING.

The innate immune system utilizes germ line-encoded pattern recognition receptors (PRRs) to detect pathogen associated molecular patterns (PAMPs) and initiate immune defense responses that counteract viral infection ^[1]^. The discovery of the multiplicity of both cytosolic RNA and DNA sensing pathways has revealed an unexpected complexity of the host response to viral nucleic acids. Detection of viral nucleic acids within the cytoplasm via either the RIG-I-MAVS signaling axis or the cGAS-STING DNA sensing pathway leads to the production of antiviral interferons (IFNs) and pro-inflammatory cytokines ^[2]^. While functioning distinctively, the DNA and RNA sensing mechanisms overlap at multiple levels. For instance, cytosolic sensing of DNA has been shown to mediate the activation and expression level of RIG-I ^[3, 4]^. Recently, new roles of the cytosolic DNA sensor cGAS in the innate control of RNA viruses has been revealed ^[5]^. In turn, there are indications that RNA sensing pathways may modulate host responses against DNA infections ^[6]^. Recently, Liu *et al*. reported that stimulation of RIG-I using a specific 5′pppRNA agonist induces STING expression at both the mRNA and protein levels. Physiologically, activation of the RIG-I-MAVS pathway efficiently suppresses infection by the DNA virus herpes simplex virus 1 (HSV-1), both *in vitro* and *in vivo* in a STING-dependent fashion ^[7]^. This study provides new insights into the cross talk between DNA and RNA pathogen-sensing systems via the control of STING.

The signaling mechanism of the cytosolic innate immunity has been extensively studied and tremendous advances have been made recently ^[2]^. STING is an important regulator in many aspects of cytosolic DNA-triggered innate immune responses. cGAS has been established as the universal sensor for cytosolic DNA from DNA viruses, bacteria, and retroviruses ^[8–11]^. Once activated by direct DNA binding, it catalyzes the production of the second messenger, cGAMP, which binds and activates STING ^[12]^. Aside from functioning as an adaptor downstream of cGAS and many other putative DNA sensors ^[13–16]^, STING also independently senses bacterial cyclic dinucleotides (CDNs) ^[17]^. The RNA cytosolic surveillance pathways involve the family of RIG-I like receptors (RLR). Among which, RIG-I specifically recognizes short dsRNA bearing a 5′ end diphosphate or triphosphate group (5′pp or 5′ppp) ^[18, 19]^. Upon viral RNA recognition, RIG-I recruits the adaptor protein MAVS which triggers a series of signaling cascades leading to the activation of the transcription factors NF-κB, IRF3, and IRF7 ^[20]^. An increasing body of evidence suggests that STING has a critical role in signaling pathways responding to RNA viral infections ^[21–23]^. However, compared to the general requirement of STING in cytosolic DNA pathogen sensing, the mechanisms of STING modulation of RNA viral sensing remain less clear. Nevertheless, both DNA- and RNA-triggered innate immune responses converge at the STING-TBK1-IRF3 axis. Activated STING recruits and activates TBK1, leading to the activation of the IRF transcription factors and NF-kB, which triggers the induction of type I IFN and inflammatory cytokines ^[1]^.

Previous work from Goulet *et al*. observed an increase in STING gene expression following RIG-I agonist stimulation in a microarray analysis ^[24]^. In the present study, Liu *et al*. validated this finding and further characterized the mechanism of RIG-I-mediated STING induction. To start, STING expression was observed to be induced by Sendai virus (SeV), a negative-sense single stranded RNA virus, among different cell types both at the protein and mRNA levels. In addition, *in vivo*, the authors detected an up-regulation of STING in the lung, liver, and spleen of C57BL/6 mice after RIG-I agonist 5′pppRNA stimulation. By employing various signaling deficient cell lines, SeV-mediated STING upregulation was shown to be exclusively activated by RIG-I. In addition, both MAVS-deficient mice and IFNAR-deficient mice displayed lower STING levels following 5′pppRNA inoculation. A detailed characterization of STING mRNA and protein expression following the 5′pppRNA treatment revealed significant increases at 24 hrs and 48 hrs in A549 epithelial cells. Altogether, these results suggest that RIG-I signaling leads to STING induction, and STING belongs to the group of late RIG-I-inducible genes.

The authors hypothesized that this delay resulted from the secretion of regulatory factors into the supernatant. Indeed, the incubation of fresh cells with the supernatants of 5′pppRNA treated cells triggered STING induction. In agreement with other publications demonstrating STING as an ISG ^[25]^, IFN-α treatment alone increased STING expression levels. More surprisingly, a remarkably high level of STING induction was achieved, not by the addition of a mixture of type I and type III IFNs, but by the co-stimulation of TNF-α and type I IFNs. Notably, the knockdown of TNFR and IFNα/βR, although significant, did not diminish the 5′pppRNA triggered STING expression completely, suggesting the possibility of other cytokine(s) driven STING induction. These results reinforced the notion that the antiviral response is not potentiated by cytokines acting independently, but rather simultaneously and synergistically. In fact, the synergistic activity of type I IFN and TNF-α has long been reported to elicit distinct antiviral states defined by a panel of late genes ^[26]^. The authors further identified the mechanisms involved in IFN- and TNF-α-dependent induction of STING. They observed a significant decrease of STING expression when transcription factors STAT1, STAT2, and the RELA subunit of NF-κB were depleted. This data suggests that synergistic TNF-α and type I IFN induction of STING expression is mediated by the convergence of the STAT and NF-κB pathways.

Since STING is a key element in the establishment of antiviral states, the authors evaluated its contribution to the immune response triggered by 5′pppRNA. The knockdown of STING using siRNA significantly sustained the expression of immune response genes including *IFNB1*, *IRF7*, *TNFAIP3*, *DDX58*, and *IFIT1* at 72 h and 96 h. Given that 5′pppRNA stimulation could protect cells against a wide range of DNA, RNA and retroviruses including vaccinia virus, influenza virus, hepatitis C virus, dengue virus, chikungunya virus and HIV-1 ^[24, 27]^, the authors next investigated whether the STING-mediated persistence of immune response gene expression has any contribution to the later time point viral resistance. Their data showed that in the 5′pppRNA pretreated cells, the HSV-1 replication status at 48 h of infection is inversely proportional to the expression level of STING. In other words, 5′pppRNA treated cells were significantly resistant to HSV-1 in the presence of STING. Correspondingly, those cells also displayed stronger immune responses at later times of HSV-1 infection. In contrast, the antiviral effect of 5′pppRNA in the context of an RNA viral infection was shown to be independent of STING. Taken together, the authors demonstrated that STING plays an active role in the 5′pppRNA-mediated restriction of HSV-1.

Furthermore, the authors verified the contribution of STING during the 5′pppRNA-mediated HSV-1 protection *in vivo*. In agreement with other publications, STING deficient mice were significantly less likely to survive HSV-1 infection ^[28]^. C57BL/6 mice weight loss was significantly reversed when pre-treated with 5′pppRNA, and were 100% protected from lethal infection with HSV-1. On the contrary, none of the STING deficient mice were rescued by 5′pppRNA pre-treatment, suggesting the vital role of STING during HSV-1 infection. Meanwhile, the authors detected in the STING deficient mice a remarkably lower serum level of IFN-β and correspondently higher viral loads in the lungs compared to the control mice. This data well correlated to the authors’ *in vitro* demonstrations and strengthened their findings on the necessity of STING during the 5′pppRNA protection against HSV-1 infection.

In summary, Liu *et al*. have demonstrated that RIG-I-mediated STING up-regulation is vital for 5′pppRNA protected HSV-1 infection both *in vitro* and in *vivo*. While this study has provided new insight into the mechanisms involved in the regulation of STING, there are still several important questions which remain to be addressed in future studies. A recent study has discovered a STAT1 binding site in the promoter region of STING as critical for its induction by IFNs ^[25]^. Does the TNF and IFN synergy induce STING expression at the transcriptional level through STAT1 and/or NF-κB binding sites in the STING promoter? In addition, since IL-1β, another NF-κB activator, failed to synergize with IFN for STING induction, it seems quite possible that other regulatory factors are involved. It would also be interesting to investigate the effects of STING-sustained interferon and the inflammatory response in the host innate immune defense against other DNA, RNA, bacterial pathogens, as well as in inflammatory disorders and autoimmune diseases. In regards to the crosstalk between DNA and RNA sensing, what are the potential roles of RNA sensing mechanisms in the activation of immune responses for the control of DNA pathogen infections? How is the DNA immune sensing involved in regulating the expression of molecules in RNA sensing pathways? Could pathogen RNAs be recognized by cytosolic DNA receptors? Further investigations on some of these topics may elucidate the complexities of the regulation of STING during innate immune signaling and provide new insight into the homeostatic control of host antiviral immune responses.

## Abbreviations

5′pp: 5′ diphosphate
5′ppp: 5′ triphosphate
α: alpha
β: beta
c-di-AMP: cyclic dimeric adenosine monophosphate
CDNs: cytosolic DNA and cyclic di-nucleotides
cGAMP: cyclic GMP-AMP
cGAS: cyclic GAMP synthase
HIV-1: human immunodeficiency virus 1
HSV-1: herpes simplex virus type 1
IFN-α: interferon alpha
IFNα/βR: IFN alpha/beta receptor
IFNAR: IFN alpha receptor
IL-1β: *interleukin 1* beta
IRF: transcription factor IFN regulatory factor
ISG: interferon stimulated genes
κ: kappa
MAVS: mitochondrial antiviral-signaling protein
NF-κB: nuclear factor-kappa B
PAMPs: pathogen-associated molecular patterns
PRRs: pattern recognition receptors
RIG-I: retinoic acid-induced gene
RLR: RIG-I like receptors
SeV: Sendai virus
siRNA: small interfering RNA
STING: stimulator of IFN genes
STAT: signal transducer and activator of transcription
TBK1: TANK binding kinase 1 complex
TNF-α: tumor necrosis factor α
TNFR: tumor necrosis factor receptor
